# The role of nuclear receptor E75 in regulating the molt cycle of *Daphnia magna* and consequences of its disruption

**DOI:** 10.1371/journal.pone.0221642

**Published:** 2019-08-27

**Authors:** Stephanie M. Street, Stephanie A. Eytcheson, Gerald A. LeBlanc

**Affiliations:** Department of Biological Sciences, North Carolina State University, Raleigh, North Carolina, United States of America; Leibniz Institute on Aging - Fritz Lipmann Institute (FLI), GERMANY

## Abstract

Biological rhythms regulate innumerable physiological processes, yet little is known of factors that regulate many of these rhythms. Disruption in the timing of these rhythms can have devastating impacts on population sustainability. We hypothesized that the timing of the molt infradian rhythm in the crustacean *Daphnia magna* is regulated by the joint action of the protein E75 and nitric oxide. Further, we hypothesized that disruption of the function of E75 would adversely impact several physiological processes related to growth and reproduction. Analysis of mRNA levels of several genes, involved in regulating the molt cycle in insects, revealed the sequential accumulation of *E75*, its dimer partner *HR3*, *FTZ-F1*, and *CYP18a1* during the molt cycle. Exposure to the nitric oxide donor sodium nitroprusside early in the molt cycle had no effect on *E75* or *HR3* mRNA levels, but delayed the peak accumulation of *FTZ-F1* and *CYP18a1* mRNA. The subsequent exuviation was also delayed consistent with the delay in peak accumulation of *FTZ-F1* and *CYP18a1*. These results supported our assertion that nitric oxide binds E75 rendering it incapable of binding HR3. Excess HR3 protein then enhanced the accumulation of the downstream products FTZ-F1 and CYP18a1. Similarly, suppression of *E75* mRNA levels, using siRNA, had no effect on mRNA levels of *HR3* but elevated mRNA levels of *FTZ-F1*. Consistent with these molecular responses, the suppression of E75 using siRNA increased the duration of the molt cycle and reduced the number of offspring produced. We conclude that the molt cycle of daphnids is regulated in a manner similar to insects and disruption of E75 results in a lengthening of the molt cycle and a reduction the release of viable offspring.

## Introduction

Biological rhythms regulate the timing of many physiological processes, and often reflect organismal adaptation to rhythmic changes in the environment [1]. Infradian rhythms are chronobiological cycles that are greater than 24 hours in length. Infradian rhythms can span from a few days to over a century long. The twenty-eight-day menstrual cycle in humans, yearly migratory and breeding patterns in animals, as well as thirteen or seventeen-year cicada emergence events are all examples of infradian rhythms [2–4].

Little is known about the molecular events involved in most infradian rhythms. The involvement of environmental cues in the entrainment of such rhythms are difficult to discern, as there does not seem to be one central regulatory clock for these rhythms, unlike with circadian rhythms [5]. Twenty-four hour circadian rhythms are typically entrained by light:dark cycles [6]. Many environmental cues seem to dictate the timing of infradian rhythms including, but not limited to, temperature, photoperiod, and food availability [7]. The range of potential entraining cues and the long timeframe of many of these rhythms makes their study challenging, but their ubiquitous nature underscores their importance to life and the regulation of biological processes.

Daphnids are crustaceans that are important constituents of freshwater zooplankton communities. Daphnids are highly responsive to environmental cues to regulate various aspects of their physiology [8]. The molt cycle in crustaceans consists of an environmentally entrained infradian rhythm [9]. This rhythm is closely aligned with physiological processes critical to population sustainability such as growth, maturation, and reproduction [10–12]. Adult *Daphnia magna* shed their exoskeleton (molt) every 3–4 days at 20°C and a 16:8 hour light:dark cycle. Molting and reproduction are synchronized endocrine-regulated processes in this species, with brood release occurring just prior to molting. Molting also allows for growth to occur. Accordingly, elucidating the molecular events that dictate the molt rhythm will enhance our understanding of the susceptibility of the rhythm to environmental influences.

**Fig 1:**
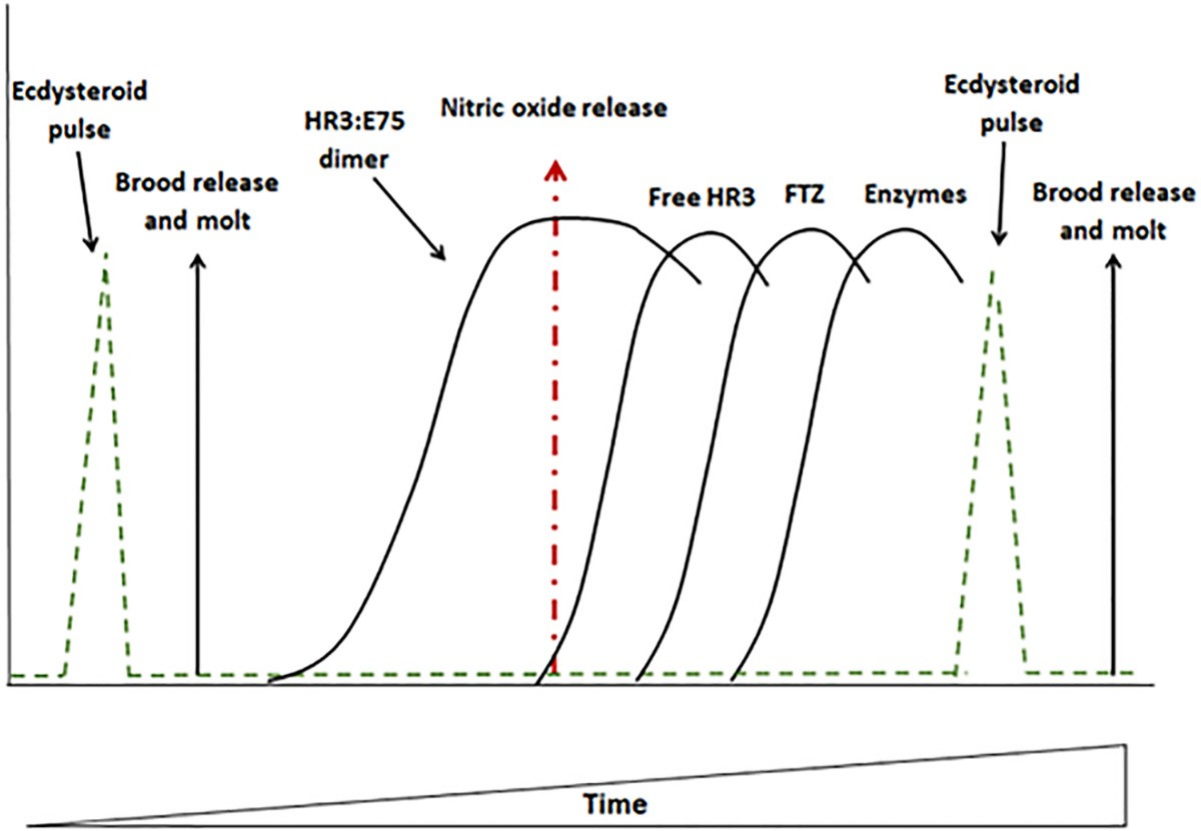
Proposed model for the regulation of the molt cycle duration by the sequential expression of nuclear receptors, nitric oxide, and enzymes involved in 20-hydroxyecdysone synthesis and inactivation in *Daphnia magna*. The pulse of 20-hydroxyecdysone at the initiation of the molt cycle induces transcription of the *E75* and *HR3* genes. Resulting HR3 and E75 proteins dimerize and progression of the cycle ceases. Nitric oxide is produced in response to environmental cues (nutrition, temperature, etc.) that influence the required time for embryo development during the molt cycle. The nitric oxide binds to E75 causing the release of HR3 protein and progression of the cycle continues. Free HR3 protein then initiates the transcription of the *FTZ-F1* gene and resulting FTZ-F1 protein initiates the transcription of enzymes responsible for the next 20-hydroxyecdysone pulse designating the end of the molt cycle and the initiation of the next cycle within the rhythm.

Previous studies conducted with various arthropod species provide a plausible hypothesis for the involvement of a gene expression cascade and nitric oxide in the regulation of the molt rhythm in daphnids. According to our model (Fig 1), the molt cycle in daphnids is initiated by a pulse of 20-hydroxyecdsone [13,14]. 20-Hydroxyecdysone induces the expression of two nuclear receptors E75 and HR3 [15]. The two nuclear receptors, E75 and HR3, can spontaneously dimerize, with E75 negatively regulating HR3’s action as a transcription factor for downstream genes [16]. E75 is a thiolate hemeprotein and has been shown to be responsive to nitric oxide [17,18]. We propose that at the appropriate time in the cycle, nitric oxide synthase produces nitric oxide which binds to E75, releasing HR3 from E75 [18–20]. Free HR3 then regulates genes involved in orchestrating the synthesis of the next pulse of 20-hydroxyecdysone which denotes the end of one cycle and the beginning of the next. Nitric oxide, we propose, regulates the duration of each cycle within the molt rhythm through its manipulation of E75’s function to ensure that the intermolt duration is timed appropriately to ensure proper development of embryos in the brood chamber. We tested aspects of this hypothesis in the present study by evaluating the sequence of expression of putative genes involved in regulating the molt rhythm and the ability of the nitric oxide donor sodium nitroprusside to alter this rhythm. We also investigated whether the targeted suppression of E75 using RNAi would elicit molecular and physiological consequences consistent the action of the nitric oxide donor.

## Materials and methods

### Daphnid cultures

*D*. *magna* used in these experiments were derived from cultures maintained at North Carolina State University. Cultures were housed in incubators at 20°C and 16:8 hour light:dark cycle as described previously [21]. These conditions favor parthenogenetic reproduction over sexual reproduction by this species. Daphnids were housed in 1 L beakers and fed 1.4x10^7^ cells of green algae (*Raphidocelis subcapitata*) and ~4 mg dry weight of Tetrafin ^®^ (Tetra, Spectrum Brands, Blacksburg, VA) fish food suspension twice daily. Fish food suspension was prepared as described previously [22]. Daphnid experiments and cultures used daphnid media composed of deionized water reconstituted with 192 mg/L CaSO_4_⋅H2O, 192 mg/L NaHCO_3_, 120 mg/L MgSO_4_, 8.0 mg/L KCl, 1.0 μg/L selenium, and 1.0 μg/L vitamin B_12_.

### Gene expression during the molt cycle

Daphnids (<24 hours old) were reared in 1L beakers for 9 days and fed as described for cultures. On day 9, individual daphnids were placed into individual 50 mL beakers with 40 mL daphnid media and fed 1.4x10^6^ cells of green algae (*R*. *subcapitata*) and ~0.4 mg dry weight of fish food suspension daily. Beginning on day 10, beaker contents were evaluated every two hours for the presence of a molted exoskeleton. The time at which the exoskeleton was observed was designated as time zero for that individual and the daphnid was randomly assigned to a time point within the molt cycle at which it would be collected. Collection times occurred at 0, 12, 36, 48, 60, 72, 78, and 84 hours post-molt. Five daphnids from the same treatment and collected at the same time point were stored together in 100 μL RNAlater (Invitrogen, Carlsbad, CA) in a 1.7 mL tube, representing one sample. Three replicate samples were collected per time point for a total of 15 daphnids per time point. Samples were held for 24 hours at 4°C and then stored at -80°C until used for RNA extraction. The duration of the molt cycle was also determined in a group of daphnids not sampled for mRNA analyses by assessing the numbers of hours between one molt and the next.

### Sodium nitroprusside treatment

Experiments were performed to disrupt the timing and duration of the putative pulse of nitric responsible for the dissociation of E75 and HR3. Daphnids were exposed to 12 μM of the nitric oxide donor, sodium nitroprusside, for 8 hrs beginning at 36 hrs post molt. This dosing regimen approximated the maximum exposure that had no discernible effect on survival or overall behavior of the organisms. According to the proposed model, the pulse of nitric oxide would normally occur after the induction of HR3. Thus, the sodium nitroprusside treatment would cause a premature and prolonged pulse of nitric oxide within the organisms resulting in the excess accumulation of free HR3. Animals were collected and analyzed for gene expression and duration of the molt cycle as described above.

### mRNA preparation and analyses

Frozen daphnids were homogenized with a bullet blender (Next Advance, Inc, Troy, NY) then RNA was extracted using the SV Total RNA Isolation System (Promega, Madison, WI). RNA concentration was measured by absorbance at 260 nm and purity was confirmed by the 260/280 nm absorbance ratio (~2.0). cDNA was prepared using ImProm-II ^™^ Reverse Transcription System (Promega) with oligo (dT) primers.

Targeted mRNAs were measured using quantitative RT-PCR. Primer preparation for *D*. *magna Actin*, *GapDH*, *E75*, *HR3*, and *FTZ-F1* have been described previously [15,23]. Primers for *D*. *magna Neverland 2*, *CYP314a1*, and *CYP18a1* were derived from the literature [24] and primers *CYP307a1*, *CYP306a1*, *CYP302a1*, and *CYP315a1* were designed based upon sequences available in wFleaBase (http://wfleabase.org/) (Table 1). The sequences were amplified with an iCycler Thermal Cycler (Bio-Rad, Hercules, CA) using 2X PCR Mastermix (Promega) at 95°C for 1 min, followed by 35 cycles with each cycle consisting of 30 sec at 95°C, 30 sec at 55°C, and 30 sec at 72°C. The amplified DNA fragments were cloned into the pCR pGEM-T Easy vector (Promega) or 4-TOPO vector (Invitrogen, Carlsbad, CA) following the manufacture’s protocol. Plasmid DNA was sequenced by Eton Bioscience Inc. (San Diego, CA) to confirm that the primers were indeed targeting the gene of interest.

**Table 1:** Oligonucleotide primers used to amplify indicated gene products by quantitative real-time PCR.

Gene	Forward	T_m_	Reverse	T_m_	Amplicon (bp)	GenBank Accession or wFleabase scaffold
*β-actin*	5’TGGTCAGGTCATCACCATTG	54.8	5’CTCGTGGATACCGCAAGATT	54.8	97	AJ292554
*GapDH*	5’GGCAAGCTAGTTGTCAATGG	54.1	5’TATTCAGCTCGAGCAGTTCC	54.5	89	AJ292555
*E75*	5’TCCGGAGAAGTATTCAACAAAAGA	54.1	5’TGCGAAGAATGGAGCACTGT	57.0	72	EF369510.1
*HR3*	5’AGTCATCACCTGCGAGGGC	60.0	5’GAACTTTGCGACCGCCG	57.5	51	FJ755466
*FTZ*-*F1*^a^	5’ATCGTGCAAGGGATTCTTCA	54.2	5’ATCAGCGACGCAAGAATAGG	55.0	63	LC105701
*CYP18a1*	5’TACCCGATCGTCGGTTACCT	57.2	5’GAGCGCCGTCAGCTCTTC	58.8	63	AB839173
*Neverland 2*	5’CGTCGGTGACTGCATCGA	57.5	5’TGCCGTCGTTCCCATTG	55.8	59	AB839172
*CYP307a1* (*spook)*	5’CAGGGCTATGCTGTCGATTT	55.1	5’CTCGTCGATGATCGTCTTGA	54.1	120	Scaffold02011
*CYP306a1* (*phantom*)	5’CGGACTGGAACGGAGAAAATGGTCCGGTT	65.2	5’AATCTCGATCCGACCTTGTGGAGCGATCCG	65.7	152	Scaffold03376
*CYP302a1* (*disembodied*)	5’AATTCAGCTGGTGAGGCAGTTCCGTATCGA	64.2	5’TGTTCGCTCGATGAAATTGAATTTCAATGG	58.4	155	Scaffold01863
*CYP315a1* (*shadow*)	5’CAACCTCGCCACTGATTCTT	55.2	5’CCAACAGGGAAGCATGAAAT	53.3	121	Scaffold00687
*CYP314a1* (*shade*)	5’GACTGCTGAAGGCGTTGACA	57.9	5’CGGCTGCCACTAGGTCGATA	58.9	62	AB257771.1

^a^The primers amplified both splice variants of *FTZ-F1*.

PCR was performed, following the manufacturers protocol, using the ABI PRISM^®^ 700 Sequence Detection System with iTaq Universal Sybr Green Supermix (BioRad, Hercules, CA) in 96-well plates (Olympus Plastics, Genesee Scientific, SanDiego, CA) sealed with ThermalSeal (Excel Scientific, Inc., Victorville, CA). mRNA levels were calculated from the raw data using GenEx software (Applied Biosystems) where the geometric mean of the Actin and GAPDH gene products were used for normalization [25]. The melting temperature of PCR products were determined using the dissociation protocol provided by the instrument manufacturer. A single melting peak was detected for all samples indicating no amplification of non-target DNA. Amplification efficiencies were >85%.

### dsRNA preparation

Oligonucleotide primers were designed to produce dsRNA targeting *D*. *magna* E75. Primers (forward: 5': TACTAATCTAGAGCGATGTCGCTATCCTCGGG, reverse: 5': TTAGTAGGTACCTTTGGTGGTTATAGTTCCAG) were synthesized (Integrated DNA Technologies, Inc. (Coralville, IA)) flanked by the XbaI and KpnI restriction enzyme sites. Each primer was amplified with the restriction enzyme sites according to the Phusion Hot Start II High-Fidelity PCR Master Mix protocol (ThermoFisher Scientific, Waltham, MA). The amplified target sequence (GCGATGTCGCTATCCTCGGGGGTCGTCAGTTCATT

GACGTCGACCCATTCAACGCTGGCCCGCTCTCTGATGGAAGGCCCTAAAATGGTTAGCACTGAACAGCAGCGCCGCGCCGATCTCATCGTCGCCAACATAATGAAGGGCAATGTTTCGGCATCGTCACCCACTCCTTCCTCATCATCGTCCGGCCAAAATTATTCAATGTCTCCGTCACCTCATTCCAGCAACAACGGACAACAGCAGAGGACCGTTCTGACGTCTGGTCCACTCTATGTCGGCTCACCCGCGCCATCTGGCAGTAGCAGCTGGAACTATAACCACCAAA) was double digested and ligated with the L4440 plasmid vector, a gift from Dr. Andrew Fire (Addgene plasmid #1654). The L4440 construct was then transformed into GC5 cells and plated on LB plates containing ampicillin (100 μg/mL, Sigma-Aldrich Corp., St. Louis, MO) and tetracycline (12.5 μg/mL, Sigma-Aldrich). Individual colonies were selected, and PCR was performed to identify inserts where ligation into the vector was successful. Successfully ligated inserts were sequenced (Eton Bioscience, San Diego, CA), and constructs were transformed into competent HT115 (DE3) cells obtained from the *Caenorhabditis* Genetics Center (funded by NIH Office of Research Infrastructure Programs (P40 Od010440)). Following transformation of HT115 cells containing the construct, stocks were prepared by growing the bacterial cells to OD_595_ = 0.040 (2.84x10^8^ cells/mL) and freezing with 25% glycerol at -80°C.

The transformed cells were grown overnight in LB medium containing ampicillin and tetracycline for use in E75 RNAi experiments. Isopropyl β-D-1-thiogalactopyranoside (Sigma-Aldrich) was added at a concentration of 2.0 mM to induce the T7 RNA polymerase and subsequent production of dsRNA of the target sequence. The concentration of transformed bacterial cells was determined by measuring the culture’s optical density at 595 nm [26]. Cells were concentrated by centrifuge at room temperature for 10 minutes at 3000 rpm, LB medium was decanted, and cells were resuspended in daphnid medium. The concentration of cells was determined according to a standard curve, and the volume required to feed 7.2×10^7^ cells/100 mL of medium was calculated.

### Suppression of E75 via RNAi

Daphnids (< 24 hours old) were used to initiate experiments aimed at the suppression of E75. Individual daphnids were isolated in 40 mL of media and fed 2.88×10^7^ cells of *E*. *coli* containing either the empty L4440 vector (no dsRNA insert) or containing the L4440 vector with the insert targeting *E75*. Daphnids were also provided 3.5×10^5^ cells of green algae (*R*. *subcapitata*) as a nutrition source. Daphnids were evaluated every two hours for the presence of a molted exoskeleton beginning fourteen days after initiation of feeding the daphnids *E*. *coli*. The first observed molt was designated time zero. Daphnids were then continually evaluated until their second molt in order to determine the length of the molt cycle, the number of offspring released, and the presence of developmental abnormalities among neonates.

Some daphnids were collected and stored in RNAlater after 14 days of feeding for mRNA preparation and analyses as described above. Three groups, with 3 daphnids per group, were sampled. Previous use of this method of mRNA suppression revealed that a minimum of 14 days of feeding *E*. *coli* containing dsRNA provided maximum suppression of targeted mRNA [26].

### Statistical analyses

Statistical comparisons were made using Student’s t tests upon establishing normality of the distributions (D’Agostino & Pearson and Shapiro-Wilk normality tests). Linear regressions were used to evaluate correlations between *FTZ-F1* and individual enzyme mRNA levels. All statistical analyses were performed using GraphPad Software (GraphPad Prism, La Jolla, CA) at an alpha level of 0.05.

## Results

### Gene expression cascade during the molt cycle

**Fig 2:**
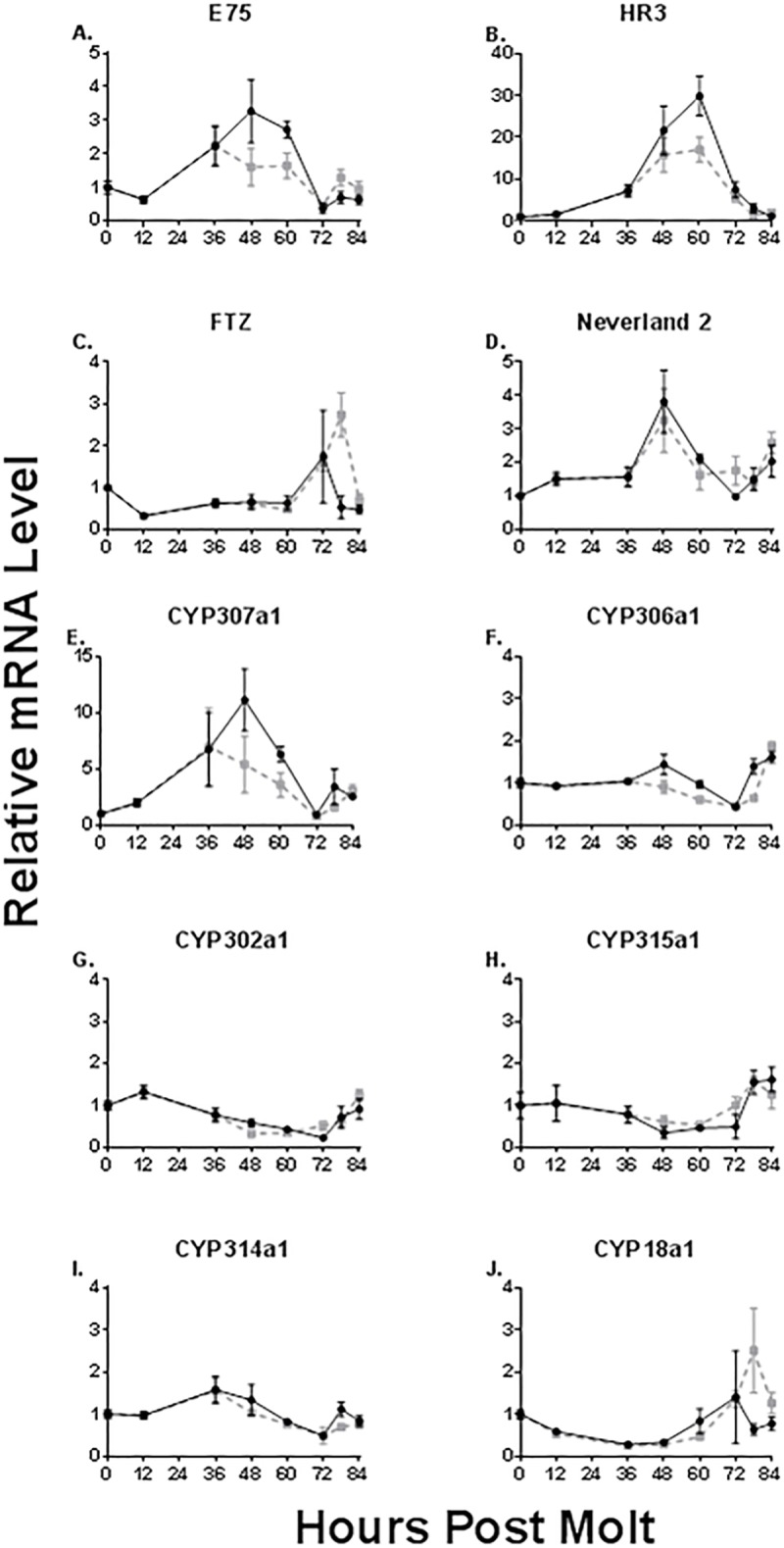
mRNA levels of relevant genes expressed during the molt cycle. Presented are temporal levels of mRNAs for *E75* (A), *HR3* (B), *FTZ-F1* (C), *Neverland 2* (D), *CYP307a1* (E), *CYP306a1* (F), *CYP302a1* (G), *CYP315a1* (H), *CYP314a1* (I) and, *CYP18a1* (J) during the molt cycle. Black solid lines represent control animals and gray dashed lines represent sodium nitroprusside-treated animals. Data are presented as mean and standard error (n = 3, with each sample consisting of mRNA from five daphnids). Raw values were normalized to their respective 0-hour value.

The temporal accumulation of mRNAs hypothesized to be involved in establishing the duration of the molt cycle was evaluated. *E75* mRNA levels attained maximum accumulation at 48 hours post-molt (Fig 2A). *HR3* mRNA levels peaked at 60 hours post-molt (Fig 2B). Maximum *FTZ-F1* mRNA levels were attained at 72 hours post-molt (Fig 2C). These results support our hypothesis that the mRNA accumulation of these nuclear receptors occurs sequentially over the molt cycle.

Next, we evaluated the mRNA accumulation profiles of the genes that contribute to the synthesis and inactivation of 20-hydroxyecdysone that ends one cycle of the molt rhythm and initiates the next. We hypothesized that the ecdysteroid synthesizing and inactivating genes were regulated by FTZ-F1 and thus would be expressed following the elevation of *FTZ-F1* mRNA levels. *Neverland 2* (Fig 2D) and *CYP307a1* (Fig 2E), the first two gene products, involved in the synthesis cascade, attained maximum accumulation at 48 hours post-molt. All subsequence mRNAs coding for biosynthetic enzymes were constitutively expressed with little evidence of increased expression during the molt cycle (Fig 2F–2I). *CYP18a1*, which is responsible for the inactivation/elimination of 20-hydroxyecdysone, mRNA levels peaked at 72 hours post-molt.

In summary, mRNA levels of the enzymes involved in 20-hydroxyecdysone synthesis did not increase after the rise in *FTZ-F1* mRNA levels indicating that these enzymes are not regulated by FTZ-F1. Rather, enzymes responsible for the initial steps in 20-hydroxyecdysone system appear to be regulated by some factor early in the cascade. Enzymes operative in the subsequent steps of 20-hydroxyecdysone synthesis appear to be constitutively expressed throughout the molt cycle. *Cyp18a1*, which is responsible for the inactivation of 20-hydroxyecdysone, mRNA levels were level commensurate with the rise in *FTZ-F1* mRNA levels suggesting that these components of the cascade may be co-regulated.

### Modulation of the signaling cascade by sodium nitroprusside

We hypothesized that nitric oxide, delivered by sodium nitroprusside, would cause the excess and prolonged release of HR3 from E75. This free HR3 then would elevate and prolong the expression of FTZ-F1 along with enzymes that are regulated by either HR3 or FTZ-F1.

Treatment with 12 μM sodium nitroprusside had no effect on the timing of the accumulation of *E75* and *HR3* mRNAs, although the magnitude of accumulation of both mRNAs was reduced (Fig 2A and 2B). Sodium nitroprusside treatment extended the accumulation of *FTZ-F1* mRNA ultimately resulting in an increase in mRNA level, as compared to the maximum accumulation in untreated daphnids, and a delay in maximum accumulation by approximately 6 hours (Fig 2C). Sodium nitroprusside had little effect on the timing or magnitude of mRNA accumulation for the enzymes involved in 20-hydroxyecdysone synthesis (Fig 2D–2I). Similar to *FTZ-F1*, *CYP18a1* mRNA accumulated to greater levels with sodium nitroprusside treatment and the timing of peak accumulation was delayed by approximately 6 hours (Fig 2J).

**Fig 3:**
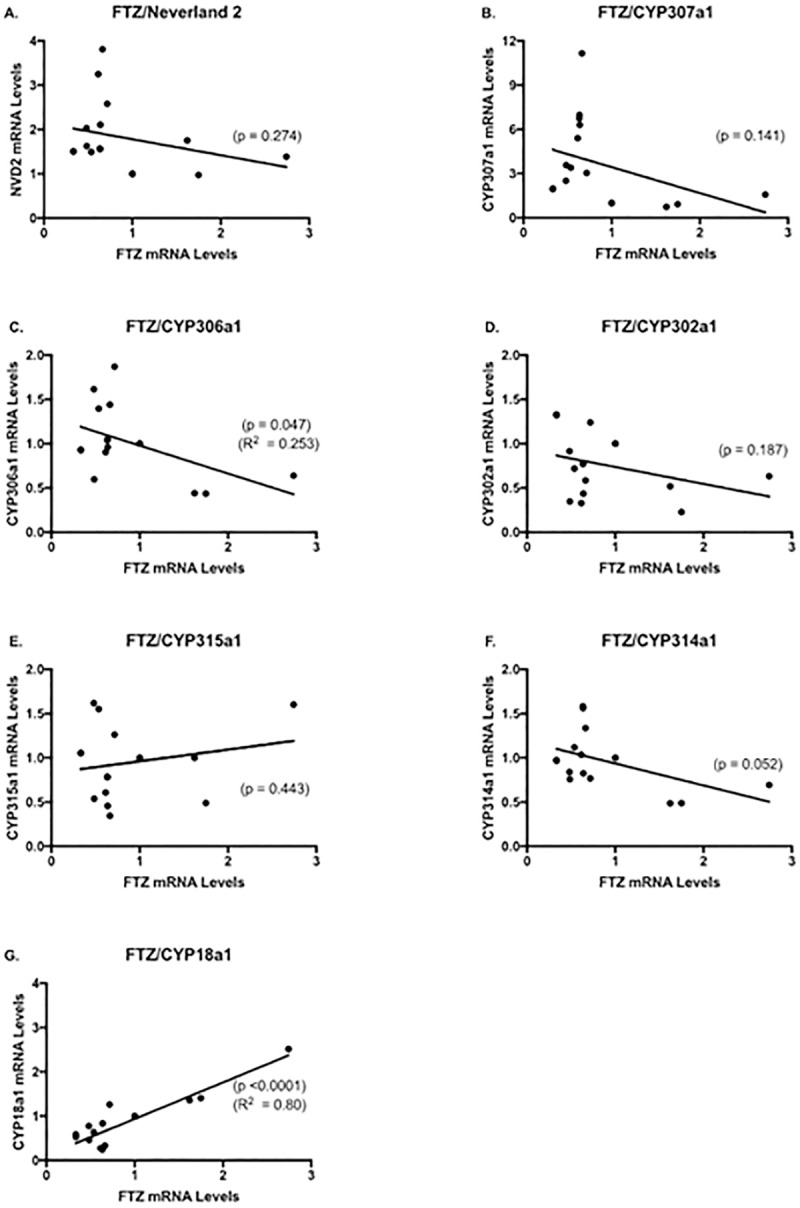
Correlation of mRNA levels for *FTZ-F1* and individual enzymes involved in the synthesis and inactivation of 20-hydroxyecdysone. Each data point represents relative mRNA levels from daphnids sampled at the same point during the molt cycle and from the same treatment (mean values, n = 3).

The relationship between mRNA levels for FTZ-F1 and individual enzymes involved in the synthesis and inactivation of 20-hydroxyecdysone during the molt cycle were evaluated in an effort to discern which, if any, of the enzymes might be regulated or co-regulated with FTZ-F1 (Fig 3). Among the gene products evaluated, only *CYP18a1* mRNA levels significantly (p<0.0001) positively correlated with *FTZ-F1* mRNA levels over the molt cycle (Fig 3G). These results support the assertion that only CYP18a1 is regulated by the E75/HR3/FTZ-F1 signaling pathway.

### Impact of sodium nitroprusside on the timing of exuviation

**Fig 4:**
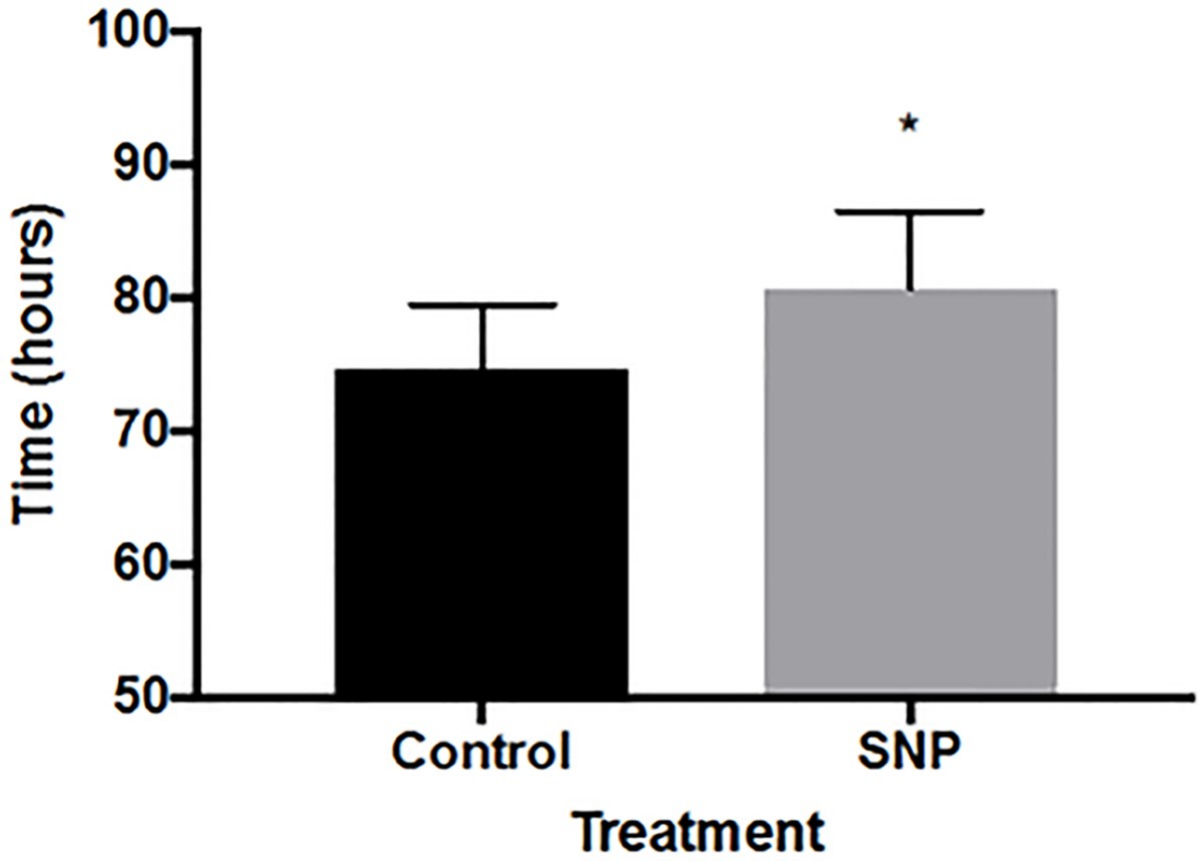
The influence of sodium nitroprusside on the duration of the molt cycle. The black bar represents control animals and the gray bar represents sodium nitroprusside (SNP)-treated animals. Data are presented as mean and standard error, n = 15 daphnids. An asterisk denotes a significant difference between control and sodium nitroprusside treated animals (p ≤ 0.05).

Shedding of the exoskeleton is the ultimate event in the molt cycle. Delays in the pulse of mRNA accumulation of *FTZ-F1* and *CYP18a1* should similarly extend the time to exuviation if these molecular events are indeed components of the molecular clock that dictates the timing of the molt rhythm. Control daphnids molted approximately 75 hours after the previous molt (Fig 4). Molting among daphnids treated with sodium nitroprusside was delayed by ~6 hours. This delay is consistent with the delay in the peak accumulation of *FTZ-F1* and *CYP18a1* mRNA.

### Suppression of E75 using RNAi

**Fig 5:**
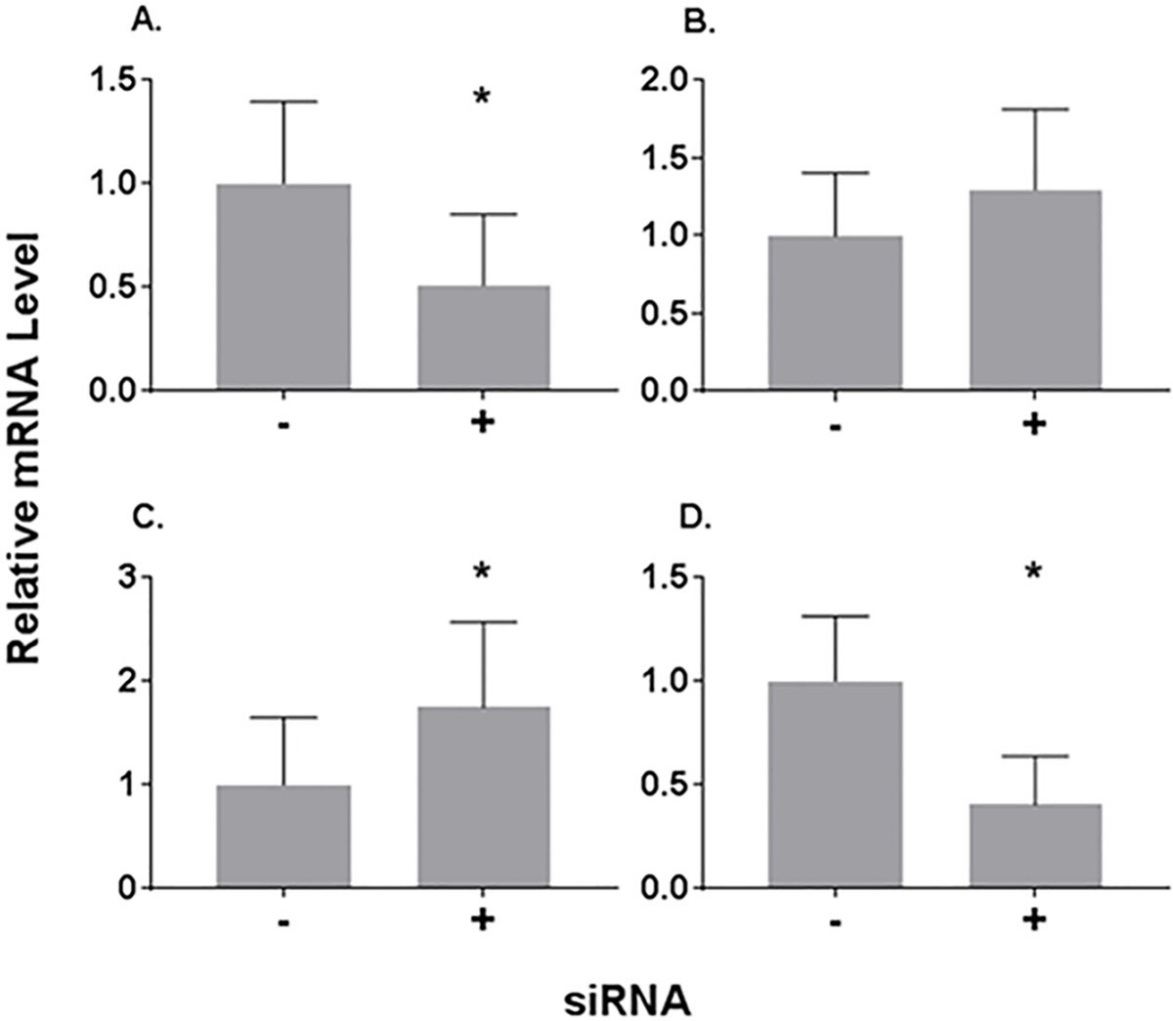
*E75*, *HR3*, *FTZ-F1*, *CYP18a1* mRNA levels with E75 dsRNA feeding. (A) *E75*, (B) *HR3*, (C) *FTZ-F1*, and (D) *CYP18a1*. Animals were sampled for mRNA analyses after 14 days of vector provision (empty vector for control, vector containing insert targeting *E75* for RNAi treatment). Sampling occurred at 36 hrs post molt. Data are presented as mean relative mRNA levels (n = 16). Error bars represent the standard errors. Significant difference (p<0.05) between treatment and control is indicated with an asterisk.

Results of experiments with sodium nitroprusside suggests that E75 is a critical determinant of the molt cycle duration. However, this conclusion is equivocal since sodium nitroprusside could be interacting with other molecular targets. Therefore, we sought to specifically inhibit E75 levels, and accordingly E75 activity, using RNAi. We hypothesized that the suppression of E75 would mimic the molecular and apical effects observed with sodium nitroprusside. Levels of *E75* mRNA were suppressed ~50% after 14 days of feeding *E*. *coli* containing E75 dsRNA (Fig 5A). Treatment with E75 dsRNA had no effect on *HR3* mRNA levels (Fig 5B). *FTZ-F1* mRNA levels increased after treatment with the E75 dsRNA (Fig 5C). This increase was consistent with the reduction of E75 protein levels causing an increase in free HR3 protein which elevated expression of FTZ-F1. *CYP18a1* mRNA levels were significantly reduced following treatment with E75 dsRNA (Fig 5D). This decrease was unexpected and may indicate that *CYP18a1* mRNA levels accumulate after FTZ-F1, where, at sampling, levels of *CYP18a1* mRNA had peaked among control daphnids but not yet in daphnids treated with E75 dsRNA.

### Apical effects of E75 suppression using RNAi

**Fig 6:**
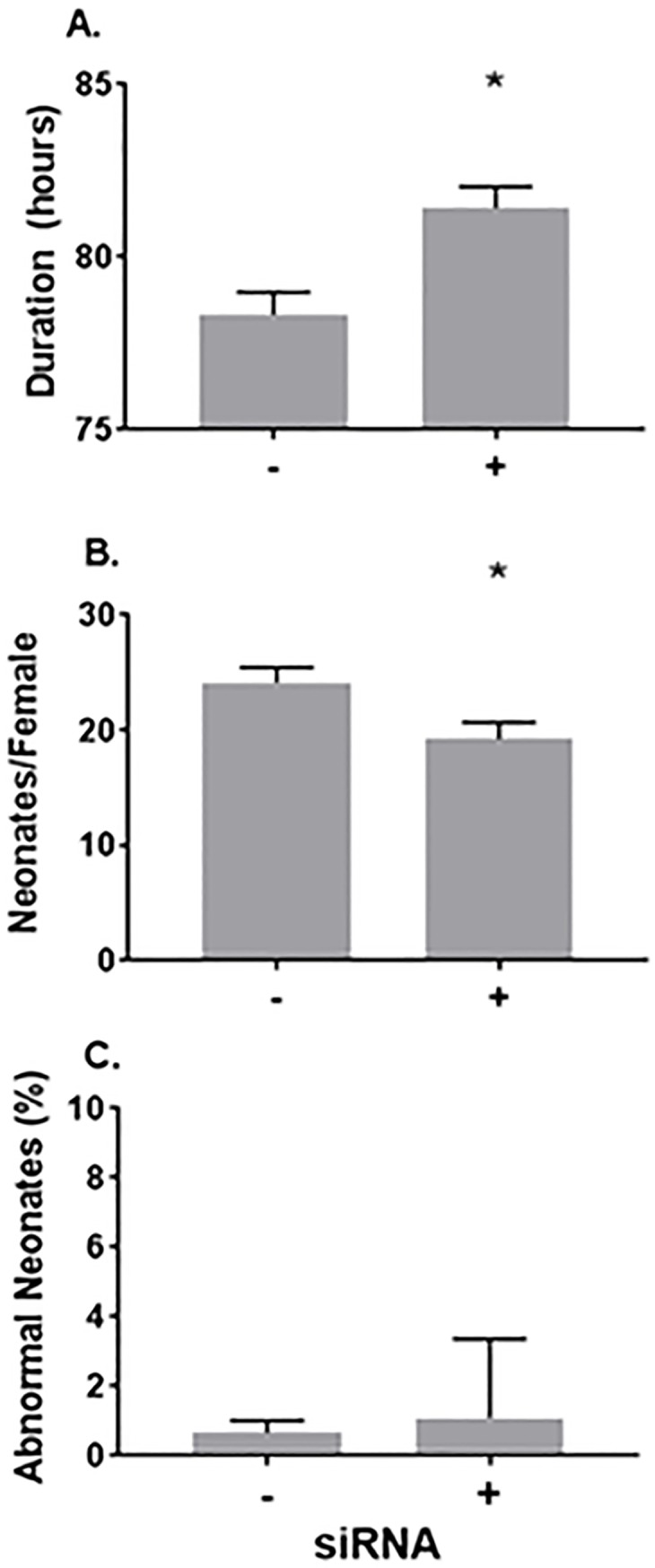
**Impact of E75 suppression on (A) the duration of the molt cycle, (B) fecundity of maternal daphnids, and (C) incidence of developmental abnormalities among offspring.** (A) Data are presented as the mean and standard error (n = 12 individual daphnids) duration of the molt cycle. (B) Data are presented as the mean and standard error (n = 19–20 individual daphnids) number of neonates per female in the first two broods. (C) Data are presented as the mean and standard error (n = 19–20 individual daphnids) percentage of abnormal neonates per maternal daphnid, determined from the same broods reported in figure B.

The targeted suppression of E75 significantly delayed molting (Fig 6A), further implicating E75 in the regulation of the daphnid molt cycle. E75 also significantly reduced the number of offspring produced by treated daphnids (Fig 6B). A low incidence of developmental abnormalities was observed in both control and E75-suppressed daphnids (Fig 6C) with no statistical significance associated with treatment.

## Discussion

Two major objectives of this study were to investigate whether gene products shown to be important in the regulation of the molt cycle in insects might also be operative in daphnids, and to investigate whether the modulation of E75 activity might serve to dictate the duration of the molt cycle.

In insects, E75 is induced by the rise in 20-hydroxyecdysone levels that occur during the last half of the molt cycle [27,28,29]. Following the induction of E75 and as ecdysteroid levels peak, HR3 production commences [27,30]. Newly produced HR3 binds to E75 protein resident in the cell. This binding to E75 prevents HR3 from acting as a transcription factor [18,20].

We previously demonstrated that HR3 is induced by exogenous 20-hydroxyecdysone administration in daphnids [15]. Herein, we demonstrated that *E75* and *HR3* mRNA levels sequentially accumulate in daphnids during the molt cycle, consistent with the expression profile of insects.

In insects, FTZ-F1 is induced by HR3 and suppressed by 20-hydroxyecdysone, thus FTZ-F1 is maximally expressed at the 20-hydroxyecdysone nadir just prior to the pulse that regulates metamorphosis [29,30,31]. FTZ-F1 expression is considered necessary to orchestrate the varied activities related to tissue-specific metamorphosis and eclosion [32]. In daphnids, *FTZ-F1* mRNA levels are elevated following the peak in *HR3* expression suggesting positive regulation by free HR3 protein. Ecdysteroid levels were not measured in the present study. Nonetheless, results suggest that FTZ-F1 is regulated in daphnids in a manner similar to that in insects.

In insects, the functionality of HR3, as a transcription factor, is suppressed by its binding to E75 [18,33]. Nitric oxide can bind to the heme moiety of E75 causing the release of HR3 [18]. HR3 can then function to regulate the expression of downstream gene involved in ecdysis including FTZ-F1 [29,30]. Similar interactions have been demonstrated among nitric oxide and the human orthologs of E75 and HR3, rev-erb and ROR, respectively [20]. We previously demonstrated that daphnid E75 is capable of suppressing the ability of HR3 to function as a transcriptional activator of a reporter gene [16]. This suppression was not the result of competition between E75 and HR3 at the response element of the regulated gene suggesting that E75 protein binds and suppresses HR3 in daphnids as observed in insects and humans.

We posited that exposure of daphnids to the nitric oxide donor, sodium nitroprusside, would block E75’s ability to bind HR3 causing a prolonged accumulation of free HR3 protein in cells. If FTZ-F1 is positively regulated by HR3, as reported in *Drosophila* [34], then we anticipated the over accumulation of *FTZ-F1* mRNA. Indeed, sodium nitroprusside caused the prolonged and increased accumulation of *FTZ-F1* resulting in a delay in its peak accumulation and a commensurate delay in molting. Similarly, provision of sodium nitroprusside to *Drosophila* delayed pupation [35]. While these results are inadequate to conclude that nitric oxide regulates the availability of free HR3 and that free HR3 regulates the expression of FTZ-F1 in daphnids, results are consistent with this hypothesis.

Maximum accumulation of *FTZ-F1* and *CYP18a1* mRNA routinely co-occurred in our experiments suggesting that these two components of the ecdysteroid signaling pathway are tightly co-regulated. In insects, *CYP18a1* is induced by the increased in 20-hydroxyecdysone levels [36]. This enzyme then functions in the inactivation of the hormone resulting the in 20-hydroxyecdysone pulse that is required for complete, successful ecdysis [37]. Daphnids similarly require the pulse of 20-hydroxyecdysone to successfully molt [38]. However, the molt cycle of daphnids is of short duration (~1–2 days in juveniles, ~3–4 days in adults) which may require novel regulation of factors involved in ecdysis. The timing of *CYP18a1* mRNA accumulation and subsequent ecdysis suggested that *CYP18a1* mRNA is expressed just prior to peak 20-hydroxyecdysone accumulation. This may be necessary to provide adequate CYP18a1 protein sufficiently early to complete the hormone pulse within the requisite time period. Thus, CYP18a1 expression may be regulated by HR3, FTZ, or some other late expression transcription factor within the ecdysteroid signaling pathway.

Importantly, enhanced accumulation of *FTZ-F1*, either through treatment with sodium nitroprusside or the suppression of E75 using RNAi, significantly lengthened the duration of the molt cycle. The molt cycle in daphnids is highly coordinated with reproduction as brood release occurs just prior to ecdysis and deposition of a new brood of embryos into the brood chamber occurs following ecdysis. Accordingly, the duration of the molt cycle must be precisely timed for the embryos to fully develop. The time required for embryos to develop can be influenced by environmental factors such as temperature [39] and light [40], thus the duration of the molt cycle must be adjusted by these factors. We proposed that the molt cycle is in a state of stasis as long as HR3 is bound to E75 and that the production of nitric oxide frees HR3 allowing the progression of the cycle to completion. Under this scenario, nitric oxide might be produced in response to environmental cues to ensure that cycle is of the appropriate duration. A molt cycle timer that is related to environmental cues has been proposed for insects [41] and nitric oxide has been proposed as a regulator of the timing of development in *Drosophila* larvae via its interaction with E75 [33]. Nitric oxide has been shown to be produced by insects in response to environmental stimuli [42].

Humans have altered the global nitrogen cycle [43,44]. The production of nitrogen-based fertilizers have significantly increased the level of reactive nitrogen as ammonia, nitrate, and nitrite. These molecules can be converted to nitric oxide through various oxidation and reduction reactions [44,45]. The binding of nitric oxide to E75 can disrupt the molt cycle of arthropods resulting in various physiological perturbations, such as growth and reproductive disturbances, that would adversely impact population sustainability. Further, the human E75 ortholog, rev-erb, has been considered as a target of drugs with the goal of adjusting circadian rhythms such as the sleep cycle [46]. The presence of such compounds in the environment, from industrial or domestic sewage discharge, at sufficient concentrations could disrupt the molt cycle of arthropods in a manner similar to nitric oxide. Diligence is warranted to ensure that such disruptions do not become a reality.

## Supporting information

S1 Dataset Supporting dataset for Figs 2–6. (PDF)
