# Supplementary material for: The role of nuclear receptor E75 in regulating the molt cycle of Daphnia magna and consequences of its disruption
Source: PLoS One. 2019 Aug 27;14(8):e0221642. doi: 10.1371/journal.pone.0221642 (PMC6711514; doi:10.1371/journal.pone.0221642)
Supplement: S1 Dataset — (PDF) [file pone.0221642.s001.pdf]

S1 Dataset. Supporting Dataset for Figs. 2-6

The Role of Nuclear Receptor E75 in Regulating the Molt Cycle of *Daphnia magna* and Consequences of its Disruption

Fig 2. mRNA levels of relevant genes expressed during the molt cycle.

C = Control, animals not exposed to SNP
T = Treatment, animals exposed to SNP at 36 hours post molt
Each data point is normalized to the mean mRNA expression of the zero hour mRNA value for that gene.
Outliers were identified by Grubb's test and were not included in calculations

E75 Control					
Identifier	Expression	Average	Standard Error	Normalized Expression	Normalized Standard Error
0 1C	2.91	4.55	0.864	1	0.19
0 2C	5.84				
0 3C	4.89				
12 C1	1.95	2.91	0.481	0.640025481	0.11
12 C2	3.36				
12 C3	3.42				
36 C 1	14.43	10.16	2.664	2.234522904	0.59
36 C 2	5.26				
36 C 3	10.78				
48 C 1	20.86	14.88	4.272	3.273066348	0.94
48 C 2	17.16				
48C 3	6.61				
60 C 1	12.43	12.38	1.109	2.723752882	0.24
60 C 2	14.28				
60 C 3	10.44				
72 C 1	2.35	1.67	0.673	0.367999383	0.15
72 C 2	1.00				
78 C 1	3.98				
78 C 2	4.04	3.20	0.810	0.703781398	0.18
78 C 3	1.58				
84 C 1	1.58				
84 C 2	3.58	2.87	0.645	0.631561083	0.14
84 C 3	3.45				

HR3 Control					
Identifier	Expression	Average	Standard Error	Normalized Expression	Normalized Standard Error
0 1C	1.00	1.16	0.079	1	0.07
0 2C	1.25				
0 3C	1.22				
12 C1	1.12	1.88	0.402	1.628802627	0.35
12 C2	2.49				
12 C3	2.04				
36 C 1	10.82	8.39	1.602	7.252377882	1.38
36 C 2	5.37				
36 C 3	8.98				
48 C 1	34.94	25.08	6.634	21.6798522	5.73
48 C 2	27.84				
48C 3	12.46				
60 C 1	27.11	34.55	5.464	29.86795295	4.72
60 C 2	31.34				
60 C 3	45.20				
72 C 1	10.80	8.70	2.100	7.519683552	1.82
72 C 2	6.60				
78 C 1	5.02				
78 C 2	1.19	3.58	1.200	3.091742807	1.04
78 C 3	4.51				
84 C 1	1.08				
84 C 2	1.32	1.31	0.127	1.130426695	0.11
84 C 3	1.52				

FTZ Control Identifier	Expression	Average	Standard Error	Normalized Expression	Normalized Standard Error
0 1C	3.79	3.97	0.096	1	0.02
0 2C	4.02				
0 3C	4.11				
12 C1	1.31	1.32	0.004	0.33193257	0.00
12 C2	1.32				
12 C3	1.33				
36 C 1	3.32	2.51	0.414	0.632931096	0.10
36 C 2	2.28				
36 C 3	1.94				
48 C 1	1.58	2.63	0.690	0.662106289	0.17
48 C 2	2.37				
48C 3	3.93				
60 C 1	2.57	2.53	0.660	0.636724159	0.17
60 C 2	3.65				
60 C 3	1.36				
72 C 1	2.57	6.95	4.374	1.748609715	1.10
72 C 2	11.32				
78 C 1	1.00				
78 C 2	4.29	2.13	1.082	0.535935546	0.27
78 C 3	1.09				
84 C 1	1.61				
84 C 2	1.46	1.91	0.376	0.480751035	0.09
84 C 3	2.66				

leverland 2 Control					
Identifier	Expression	Average	Standard Error	Normalized Expression	Normalized Standard Error
0 1C	1.44	1.60	0.135	1	0.08
0 2C	1.87				
0 3C	1.50				
12 C1	1.82	2.42	0.298	1.509949613	0.19
12 C2	2.68				
12 C3	2.75				
36 C 1	3.34	2.51	0.463	1.56938904	0.29
36 C 2	1.74				
36 C 3	2.44				
48 C 1	4.31	6.10	1.491	3.810762084	0.93
48 C 2	9.06				
48C 3	4.93				
60 C 1	3.02	3.38	0.216	2.109931595	0.13
60 C 2	3.34				
60 C 3	3.76				
72 C 1	1.59	1.56	0.032	0.976509693	0.02
72 C 2	1.53				
78 C 1	3.43				
78 C 2	2.05	2.39	0.532	1.494796948	0.33
78 C 3	1.69				
84 C 1	2.73				
84 C 2	4.74	3.25	0.754	2.033062272	0.47
84 C 3	2.29				

CYP307a1 Control

Identifier	Expression	Average	Standard Err	Normalized Expressi	Normalized Standard Error
0 1C	1.06	1.12	0.082	1	0.07
0 2C	1.02				
0 3C	1.28				
12 C1	1.43	2.19	0.398	1.955451446	0.36
12 C2	2.36				
12 C3	2.78				
36 C 1	14.62	7.56	3.729	6.746234094	3.33
36 C 2	6.10				
36 C 3	1.96				
48 C 1	10.15	12.52	3.093	11.16892419	2.76
48 C 2	18.65				
48C 3	8.75				
60 C 1	6.07	7.06	0.757	6.302128688	0.68
60 C 2	6.57				
60 C 3	8.55				
72 C 1	1.07	1.04	0.037	0.925152583	0.03
72 C 2	1.00				
78 C 1	7.35				
78 C 2	1.85	3.82	1.770	3.406219919	1.58
78 C 3	2.25				
84 C 1	2.37				
84 C 2	3.26	2.82	0.259	2.513488433	0.23
84 C 3	2.82				

CYP306a1 Control						
Identifier	Expression		Average	Standard Error	Normalized E	Normalized Standard Error
0 1C		2.32	2.30	0.257	1	0.11
0 2C		1.84				
0 3C		2.73				
12 C1		2.12	2.13	0.076	0.92918516	0.03
12 C2		2.01				
12 C3		2.27				
36 C 1		5.34	2.39	0.026	1.041544	0.01
36 C 2		2.42	36 C1 outlier			
36 C 3		2.37				
48 C 1		2.24	3.31	0.535	1.44064193	0.23
48 C 2		3.89				
48C 3		3.80				
60 C 1		2.12	2.21	0.196	0.96008696	0.09
60 C 2		1.91				
60 C 3		2.58				
72 C 1		1.00	1.00	0.001	0.43581165	0.00
72 C 2		1.00				
78 C 1		4.03				
78 C 2		2.91	3.21	0.415	1.39629435	0.18
78 C 3		2.69				
84 C 1		3.13				
84 C 2		4.12	3.71	0.297	1.61580274	0.13
84 C 3		3.88				

CYP302a1 Control					
Identifier	Expression	Average	Standard Error	Normalized E	Normalized Standard Error
0 1C	4.64	5.88	0.642	1	0.11
0 2C	6.79				
0 3C	6.21				
12 C1	6.04	7.82	0.921	1.32992619	0.16
12 C2	8.29				
12 C3	9.13				
36 C 1	6.00	4.54	0.928	0.77260532	0.16
36 C 2	2.82				
36 C 3	4.80				
48 C 1	4.38	3.43	0.545	0.58404771	0.09
48 C 2	3.43				
48C 3	2.49				
60 C 1	2.83	2.56	0.182	0.43519923	0.03
60 C 2	2.63				
60 C 3	2.21				
72 C 1	1.00	1.33	0.334	0.22690305	0.06
72 C 2	1.67				
78 C 1	7.16				
78 C 2	2.53	4.22	1.473	0.718444	0.25
78 C 3	2.99				
84 C 1	7.37				
84 C 2	5.98	5.38	1.354	0.91497844	0.23
84 C 3	2.79				

CYP314a1 Control					
Identifier	Expression	Average	Standard Error	Normalized Expression	Normalized Standard Error
0 1C	1.96	2.06	0.203	1	0.10
0 2C	2.46				
0 3C	1.78				
12 C1	1.93	2.01	0.178	0.97359607	0.09
12 C2	1.75				
12 C3	2.35				
36 C 1	4.26	3.27	0.651	1.583759168	0.32
36 C 2	2.04				
36 C 3	3.51				
48 C 1	2.92	2.76	0.768	1.337282633	0.37
48 C 2	4.00				
48C 3	1.36				
60 C 1	1.73	1.70	0.032	0.825693362	0.02
60 C 2	1.64				
60 C 3	1.74				
72 C 1	1.02	1.01	0.009	0.48872407	0.00
72 C 2	1.00				
78 C 1	2.97				
78 C 2	1.76	2.31	0.352	1.11977969	0.17
78 C 3	2.21				
84 C 1	1.85				
84 C 2	1.28	1.74	0.239	0.841532341	0.12
84 C 3	2.08				

CYP18a1 Control					
Identifier	Expression	Average	Standard Error	Normalized Expression	Normalized Standard Error
0 1C	6.61	5.90	0.675	1	0.11
0 2C	4.55				
0 3C	6.55				
12 C1	2.83	3.48	0.326	0.589355213	0.06
12 C2	3.75				
12 C3	3.85				
36 C 1	1.71	1.64	0.349	0.277323732	0.06
36 C 2	1.00				
36 C 3	2.20				
48 C 1	1.96	1.97	0.012	0.333850745	0.00
48 C 2	3.32	outlier			
48C 3	1.98				
60 C 1	4.02	4.94	1.705	0.837806584	0.29
60 C 2	8.25				
60 C 3	2.57				
72 C 1	1.82	8.32	6.501	1.409893508	1.10
72 C 2	14.82				
78 C 1	5.30				
78 C 2	3.34	3.78	0.784	0.639806361	0.13
78 C 3	2.69				
84 C 1	3.76				
84 C 2	6.38	4.59	0.897	0.777124178	0.15
84 C 3	3.62				

E75 SNP Identifier	Expression	Average	Standard Error	Normalized Expression	Normalized Standard Error
0 1C	1.66	2.59	0.494	1	0.19
0 2C	3.33				
0 3C	2.79				
12 C1	1.11	1.66	0.275	0.640073657	0.11
12 C2	1.91				
12 C3	1.96				
36 C 1	8.29	5.83	1.530	2.247697208	0.59
36 C 2	3.02				
36 C 3	6.18				
48 T 1	6.95	4.16	1.436	1.605104097	0.55
48 T 2	3.38				
48 T 3	2.16				
60 T 1	3.79	4.27	0.984	1.645834962	0.38
60 T 2	6.16				
60 T 3	2.86				
72 T 1	1.00	1.20	0.117	0.461917219	0.05
72 T 2	1.19				
72 T 3	1.41				
78 T 1	3.56	3.34	0.631	1.288792233	0.24
78 T 2	2.16				
78 T 3	4.31				
84 T 1	3.42	2.47	0.499	0.953880852	0.19
84 T 2	2.58				
84 T 3	1.42				

HR3 SNP Identifier	Expression	Average	Standard Error	Normalized Expression	Normalized Standard Error
0 1C	1.29	1.49	0.101	1	0.07
0 2C	1.61				
0 3C	1.56				
12 C1	1.44	2.41	0.511	1.617060044	0.34
12 C2	3.18				
12 C3	2.60				
36 C 1	13.59	10.55	2.008	7.089881426	1.35
36 C 2	6.75				
36 C 3	11.29				
48 T 1	30.86	23.50	6.130	15.80023753	4.12
48 T 2	28.31				
48 T 3	11.33				
60 T 1	17.99	25.40	4.393	17.07714742	2.95
60 T 2	33.20				
60 T 3	25.01				
72 T 1	5.95	7.98	1.145	5.365839844	0.77
72 T 2	8.07				
72 T 3	9.92				
78 T 1	3.09	2.05	2.649	1.377303849	1.78
78 T 2	1.19				
78 T 3	1.86				
84 T 1	5.39	3.06	1.179	2.060466702	0.79
84 T 2	1.59				
84 T 3	2.22				

FTZ SNP Identifier	Expression	Average	Standard Error	Normalized Expression	Normalized Standard Error
0 1C	2.87	3.01	0.072	1	0.02
0 2C	3.05				
0 3C	3.11				
12 C1	1.00	1.01	0.003	0.33417976	0.00
12 C2	1.01				
12 C3	1.01				
36 C 1	2.52	1.91	0.314	0.634162857	0.10
36 C 2	1.73				
36 C 3	1.48				
48 T 1	2.62	1.85	0.388	0.614790598	0.13
48 T 2	1.40				
48 T 3	1.53				
60 T 1	1.65	1.46	0.096	0.483801639	0.03
60 T 2	1.35				
60 T 3	1.38				
72 T 1	6.28	4.88	0.700	1.621433841	0.23
72 T 2	4.25				
72 T 3	4.12				
78 T 1	10.38	8.26	1.592	2.742897702	0.53
78 T 2	9.26				
78 T 3	5.15				
84 T 1	2.59	2.16	0.387	0.716330292	0.13
84 T 2	2.49				
84 T 3	1.39				

Neverland 2 SNP					
Identifier	Expression	Average	Standard Error	Normalized Expression	Normalized Standard Error
0 1C	1.06	1.18	0.099	1	0.08
0 2C	1.38				
0 3C	1.11				
12 C1	1.34	1.78	0.218	1.50405721	0.18
12 C2	1.97				
12 C3	2.02				
36 C 1	2.46	1.85	0.340	1.562938731	0.29
36 C 2	1.28				
36 C 3	1.80				
48 T 1	6.04	3.85	1.123	3.252386758	0.95
48 T 2	3.18				
48 T 3	2.32				
60 T 1	1.00	1.93	0.518	1.628940931	0.44
60 T 2	2.79				
60 T 3	1.99				
72 T 1	1.47	2.08	0.506	1.756571064	0.43
72 T 2	1.68				
72 T 3	3.08				
78 T 1	2.10	1.64	0.232	1.388418698	0.20
78 T 2	1.37				
78 T 3	1.45				
84 T 1	3.34	3.05	0.381	2.583318722	0.32
84 T 2	3.53				
84 T 3	2.30				

CYP307a1 SNP

Identifier	Expression	Average	Standard Err	Normalized Expressi	Normalized Standard Error
0 1C	1.98	2.09	0.157	1	0.07
0 2C	1.90				
0 3C	2.40				
12 C1	2.71	4.15	0.765	1.983279388	0.37
12 C2	4.45				
12 C3	5.31				
36 C 1	28.37	14.63	7.261	6.981536841	3.47
36 C 2	11.81				
36 C 3	3.70				
48 T 1	21.80	11.32	5.288	5.40322244	2.52
48 T 2	7.34				
48 T 3	4.82				
60 T 1	3.74	7.49	2.295	3.576915595	1.10
60 T 2	11.66				
60 T 3	7.08				
72 T 1	1.00	1.56	0.451	0.744357204	0.22
72 T 2	1.23				
72 T 3	2.45				
78 T 1	3.66	3.29	0.330	1.571296613	0.16
78 T 2	2.63				
78 T 3	3.58				
84 T 1	5.60	6.37	1.143	3.040918654	0.55
84 T 2	8.62				
84 T 3	4.89				

CYP302a1 SNP						
Identifier	Expression	Average	Standard Error	Normalized E	Normalized Standard Error	
0 1C	3.60	4.55	0.494	1	0.11	
0 2C	5.26					
0 3C	4.80					
12 C1	4.66	6.02	0.702	1.32235034	0.15	
12 C2	6.40					
12 C3	7.00					
36 C 1	4.65	3.52	0.717	0.77291788	0.16	
36 C 2	2.19					
36 C 3	3.72					
48 T 1	1.68	1.49	0.249	0.32799039	0.05	
48 T 2	1.80					
48 T 3	1.00					
60 T 1	1.64	1.58	0.075	0.34779991	0.02	
60 T 2	1.67					
60 T 3	1.43					
72 T 1	1.59	2.36	0.473	0.51791677	0.10	
72 T 2	2.26					
72 T 3	3.22					
78 T 1	2.22	2.88	0.684	0.63291849	0.15	
78 T 2	2.18					
78 T 3	4.25					
84 T 1	6.68	5.65	0.656	1.24158258	0.14	
84 T 2	5.85					
84 T 3	4.43					

꞉YP315a1 SNP					
Identifier	Expression	Average	Standard Error	Normalized Expression	Normalized Standard Error
0 1C	4.42	2.73	0.864	1	0.32
0 2C	1.58				
0 3C	2.18				
12 C1	5.02	2.88	1.169	1.05566293	0.43
12 C2	2.61				
12 C3	1.00				
36 C 1	1.11	2.14	0.522	0.784802795	0.19
36 C 2	2.52				
36 C 3	2.79				
48 T 1	1.42	1.66	0.369	0.608985303	0.14
48 T 2	1.17				
48 T 3	2.38				
60 T 1	1.80	1.47	0.185	0.53839709	0.07
60 T 2	1.45				
60 T 3	1.16				
72 T 1	3.44	2.73	0.561	1.001149189	0.21
72 T 2	3.12				
72 T 3	1.62				
78 T 1	1.39	4.37	0.007	1.603020056	0.00
78 T 2	4.38	78 T1 outlier			
78 T 3	4.36				
84 T 1	2.71	3.44	0.932	1.26278894	0.34
84 T 2	2.33				
84 T 3	5.29				

YP314a1 Treatment					
Identifier	Expression	Average	Standard Error	Normalized Expression	Normalized Standard Error
0 1C	7.90	8.33	0.812	1	0.10
0 2C	9.90				
0 3C	7.19				
12 C1	7.73	8.07	0.676	0.968927216	0.08
12 C2	7.11				
12 C3	9.38				
36 C 1	16.93	13.01	2.582	1.561966961	0.31
36 C 2	8.14				
36 C 3	13.97				
48 T 1	8.66	8.63	0.356	1.036467765	0.04
48 T 2	9.24				
48 T 3	8.01				
60 T 1	5.63	6.33	0.350	0.759605462	0.04
60 T 2	6.71				
60 T 3	6.64				
72 T 1	1.00	4.07	1.636	0.48814803	0.20
72 T 2	4.61				
72 T 3	6.59				
78 T 1	5.14	5.78	0.361	0.693523863	0.04
78 T 2	5.80				
78 T 3	6.39				
84 T 1	5.82	6.40	0.774	0.768586162	0.09
84 T 2	5.45				
84 T 3	7.94				

CYP18a1 SNP Identifier	Expression	Average	Standard Error	Normalized Expression	Normalized Standard Error
0 1C	6.63	6.60	0.030	1	0.00
0 2C	4.55	outlier			
0 3C	6.57				
12 C1	2.84	3.49	0.326	0.528355309	0.05
12 C2	3.75				
12 C3	3.87				
36 C 1	1.70	1.64	0.349	0.247935206	0.05
36 C 2	1.00				
36 C 3	2.20				
48 T 1	2.29	1.83	0.231	0.277584373	0.03
48 T 2	1.54				
48 T 3	1.67				
60 T 1	2.80	3.06	0.432	0.464451191	0.07
60 T 2	2.48				
60 T 3	3.91				
72 T 1	11.51	8.98	1.337	1.361420525	0.20
72 T 2	6.96				
72 T 3	8.47				
78 T 1	28.72	16.62	6.674	2.519986744	1.01
78 T 2	15.47				
78 T 3	5.69				
84 T 1	10.76	8.34	1.709	1.264926284	0.26
84 T 2	9.23				
84 T 3	5.04				

Fig 3. Correlation of mRNA levels for *FTZ-F1* and individual enzymes involved in the synthesis and inactivation of 20-hydroxyecdysone.

C = Control, animals not exposed to SNP
T = Treatment, animals exposed to SNP at 36 hours post molt
Each data point is the average of the expression for each time point in both control and treatment.
Outliers were identified using Grubb's test and were not included in the calculations

E75 Control			HR3 Control		
Identifier	Expression	Average	Identifier	Expression	Average
0 1C	2.91	4.55	0 1C	1.00	1.16
0 2C	5.84		0 2C	1.25	
0 3C	4.89		0 3C	1.22	
12 C1	1.95	2.91	12 C1	1.12	1.88
12 C2	3.36		12 C2	2.49	
12 C3	3.42		12 C3	2.04	
36 C 1	14.43	10.16	36 C 1	10.82	8.39
36 C 2	5.26		36 C 2	5.37	
36 C 3	10.78		36 C 3	8.98	
48 C 1	20.86	14.88	48 C 1	34.94	25.08
48 C 2	17.16		48 C 2	27.84	
48C 3	6.61		48C 3	12.46	
60 C 1	12.43	12.38	60 C 1	27.11	34.55
60 C 2	14.28		60 C 2	31.34	
60 C 3	10.44		60 C 3	45.20	
72 C 1	2.35	1.67	72 C 1	10.80	8.70
72 C 2	1.00		72 C 2	6.60	
78 C 1	3.98		78 C 1	5.02	
78 C 2	4.04	3.20	78 C 2	1.19	3.58
78 C 3	1.58		78 C 3	4.51	
84 C 1	1.58		84 C 1	1.08	
84 C 2	3.58	2.87	84 C 2	1.32	1.31
84 C 3	3.45		84 C 3	1.52	

FTZ Control				Neverland 2 Control		
Identifier	Expression	Average		Identifier	Expression	Average
0 1C	3.79	3.97		0 1C	1.44	1.60
0 2C	4.02			0 2C	1.87	
0 3C	4.11			0 3C	1.50	
12 C1	1.31	1.32		12 C1	1.82	2.42
12 C2	1.32			12 C2	2.68	
12 C3	1.33			12 C3	2.75	
36 C 1	3.32	2.51		36 C 1	3.34	2.51
36 C 2	2.28			36 C 2	1.74	
36 C 3	1.94			36 C 3	2.44	
48 C 1	1.58	2.63		48 C 1	4.31	6.10
48 C 2	2.37			48 C 2	9.06	
48C 3	3.93			48C 3	4.93	
60 C 1	2.57	2.53		60 C 1	3.02	3.38
60 C 2	3.65			60 C 2	3.34	
60 C 3	1.36			60 C 3	3.76	
72 C 1	2.57	6.95		72 C 1	1.59	1.56
72 C 2	11.32			72 C 2	1.53	
78 C 1	1.00			78 C 1	3.43	
78 C 2	4.29	2.13		78 C 2	2.05	2.39
78 C 3	1.09			78 C 3	1.69	
84 C 1	1.61			84 C 1	2.73	
84 C 2	1.46	1.91		84 C 2	4.74	3.25
84 C 3	2.66			84 C 3	2.29	

'P307a1 Control		
Identifier	Expression	Average
0 1C	1.06	1.12
0 2C	1.02	
0 3C	1.28	
12 C1	1.43	2.19
12 C2	2.36	
12 C3	2.78	
36 C 1	14.62	7.56
36 C 2	6.10	
36 C 3	1.96	
48 C 1	10.15	12.52
48 C 2	18.65	
48C 3	8.75	
60 C 1	6.07	7.06
60 C 2	6.57	
60 C 3	8.55	
72 C 1	1.07	1.04
72 C 2	1.00	
78 C 1	7.35	
78 C 2	1.85	3.82
78 C 3	2.25	
84 C 1	2.37	
84 C 2	3.26	2.82
84 C 3	2.82	

CYP306a1 Control		
Identifier	Expression	Average
0 1C	2.32	2.30
0 2C	1.84	
0 3C	2.73	
12 C1	2.12	2.13
12 C2	2.01	
12 C3	2.27	
36 C 1	5.34	2.39
36 C 2	2.42	36 C1 outlier
36 C 3	2.37	
48 C 1	2.24	3.31
48 C 2	3.89	
48C 3	3.80	
60 C 1	2.12	2.21
60 C 2	1.91	
60 C 3	2.58	
72 C 1	1.00	1.00
72 C 2	1.00	
78 C 1	4.03	
78 C 2	2.91	3.21
78 C 3	2.69	
84 C 1	3.13	
84 C 2	4.12	3.71
84 C 3	3.88	

CYP302a1 Control		
Identifier	Expression	Average
0 1C	4.64	5.88
0 2C	6.79	
0 3C	6.21	
12 C1	6.04	7.82
12 C2	8.29	
12 C3	9.13	
36 C 1	6.00	4.54
36 C 2	2.82	
36 C 3	4.80	
48 C 1	4.38	3.43
48 C 2	3.43	
48C 3	2.49	
60 C 1	2.83	2.56
60 C 2	2.63	
60 C 3	2.21	
72 C 1	1.00	1.33
72 C 2	1.67	
78 C 1	7.16	
78 C 2	2.53	4.22
78 C 3	2.99	
84 C 1	7.37	
84 C 2	5.98	5.38
84 C 3	2.79	

CYP315a1 Control		
Identifier	Expression	Average
0 1C	8.81	5.44
0 2C	3.15	
0 3C	4.36	
12 C1	9.99	5.73
12 C2	5.22	
12 C3	1.99	
36 C 1	2.21	4.26
36 C 2	5.02	
36 C 3	5.56	
48 C 1	1.27	1.88
48 C 2	1.00	
48C 3	3.38	
60 C 1	3.03	2.49
60 C 2	2.00	
60 C 3	2.43	
72 C 1	1.13	2.67
72 C 2	4.21	
78 C 1	9.08	
78 C 2	5.51	8.44
78 C 3	10.75	
84 C 1	11.69	
84 C 2	6.33	8.81
84 C 3	8.42	

CYP314a1 Control		
Identifier	Expression	Average
0 1C	1.96	2.06
0 2C	2.46	
0 3C	1.78	
12 C1	1.93	2.01
12 C2	1.75	
12 C3	2.35	
36 C 1	4.26	3.27
36 C 2	2.04	
36 C 3	3.51	
48 C 1	2.92	2.76
48 C 2	4.00	
48C 3	1.36	
60 C 1	1.73	1.70
60 C 2	1.64	
60 C 3	1.74	
72 C 1	1.02	1.01
72 C 2	1.00	
78 C 1	2.97	
78 C 2	1.76	2.31
78 C 3	2.21	
84 C 1	1.85	
84 C 2	1.28	1.74
84 C 3	2.08	

CYP18a1 Control		
Identifier	Expression	Average
0 1C	6.61	5.90
0 2C	4.55	
0 3C	6.55	
12 C1	2.83	3.48
12 C2	3.75	
12 C3	3.85	
36 C 1	1.71	1.64
36 C 2	1.00	
36 C 3	2.20	
48 C 1	1.96	1.97
48 C 2	3.32	outlier
48C 3	1.98	
60 C 1	4.02	4.94
60 C 2	8.25	
60 C 3	2.57	
72 C 1	1.82	8.32
72 C 2	14.82	
78 C 1	5.30	
78 C 2	3.34	3.78
78 C 3	2.69	
84 C 1	3.76	
84 C 2	6.38	4.59
84 C 3	3.62	

E75 Treatment		
Identifier	Expression	Average
0 1C	1.66	2.59
0 2C	3.33	
0 3C	2.79	
12 C1	1.11	1.66
12 C2	1.91	
12 C3	1.96	
36 C 1	8.29	5.83
36 C 2	3.02	
36 C 3	6.18	
48 T 1	6.95	4.16
48 T 2	3.38	
48 T 3	2.16	
60 T 1	3.79	4.27
60 T 2	6.16	
60 T 3	2.86	
72 T 1	1.00	1.20
72 T 2	1.19	
72 T 3	1.41	
78 T 1	3.56	3.34
78 T 2	2.16	
78 T 3	4.31	
84 T 1	3.42	2.47
84 T 2	2.58	
84 T 3	1.42	

HR3 Treatment		
Identifier	Expression	Average
0 1C	1.29	1.49
0 2C	1.61	
0 3C	1.56	
12 C1	1.44	2.41
12 C2	3.18	
12 C3	2.60	
36 C 1	13.59	10.55
36 C 2	6.75	
36 C 3	11.29	
48 T 1	30.86	23.50
48 T 2	28.31	
48 T 3	11.33	
60 T 1	17.99	25.40
60 T 2	33.20	
60 T 3	25.01	
72 T 1	5.95	7.98
72 T 2	8.07	
72 T 3	9.92	
78 T 1	3.09	2.05
78 T 2	1.19	
78 T 3	1.86	
84 T 1	5.39	3.06
84 T 2	1.59	
84 T 3	2.22	

FTZ Treatment				Neverland 2 Treatment		
Identifier	Expression	Average		Identifier	Expression	Average
0 1C	2.87	3.01		0 1C	1.06	1.18
0 2C	3.05			0 2C	1.38	
0 3C	3.11			0 3C	1.11	
12 C1	1.00	1.01		12 C1	1.34	1.78
12 C2	1.01			12 C2	1.97	
12 C3	1.01			12 C3	2.02	
36 C 1	2.52	1.91		36 C 1	2.46	1.85
36 C 2	1.73			36 C 2	1.28	
36 C 3	1.48			36 C 3	1.80	
48 T 1	2.62	1.85		48 T 1	6.04	3.85
48 T 2	1.40			48 T 2	3.18	
48 T 3	1.53			48 T 3	2.32	
60 T 1	1.65	1.46		60 T 1	1.00	1.93
60 T 2	1.35			60 T 2	2.79	
60 T 3	1.38			60 T 3	1.99	
72 T 1	6.28	4.88		72 T 1	1.47	2.08
72 T 2	4.25			72 T 2	1.68	
72 T 3	4.12			72 T 3	3.08	
78 T 1	10.38	8.26		78 T 1	2.10	1.64
78 T 2	9.26			78 T 2	1.37	
78 T 3	5.15			78 T 3	1.45	
84 T 1	2.59	2.16		84 T 1	3.34	3.05
84 T 2	2.49			84 T 2	3.53	
84 T 3	1.39			84 T 3	2.30	

CYP307a1 Treatment

Identifier	Expression	Average
0 1C	1.98	2.09
0 2C	1.90	
0 3C	2.40	
12 C1	2.71	4.15
12 C2	4.45	
12 C3	5.31	
36 C 1	28.37	14.63
36 C 2	11.81	
36 C 3	3.70	
48 T 1	21.80	11.32
48 T 2	7.34	
48 T 3	4.82	
60 T 1	3.74	7.49
60 T 2	11.66	
60 T 3	7.08	
72 T 1	1.00	1.56
72 T 2	1.23	
72 T 3	2.45	
78 T 1	3.66	3.29
78 T 2	2.63	
78 T 3	3.58	
84 T 1	5.60	6.37
84 T 2	8.62	
84 T 3	4.89	

CYP306a1 Treatment

Identifier	Expression	Average
0 1C	2.32	2.29
0 2C	1.84	
0 3C	2.72	
12 C1	2.12	2.13
12 C2	2.01	
12 C3	2.27	
36 C 1	5.34	2.39
36 C 2	2.42	36 C1 outlier
36 C 3	2.36	
48 T 1	2.68	2.07
48 T 2	2.03	
48 T 3	1.51	
60 T 1	1.19	1.37
60 T 2	1.26	
60 T 3	1.66	
72 T 1	1.03	1.01
72 T 2	1.00	
72 T 3	1.00	
78 T 1	1.37	1.47
78 T 2	1.28	
78 T 3	1.75	
84 T 1	3.93	4.29
84 T 2	4.78	
84 T 3	4.16	

CYP302a1 Treatment				CYP315a1 Treatment		
Identifier	Expression	Average		Identifier	Expression	Average
0 1C	3.60	4.55		0 1C	4.42	2.73
0 2C	5.26			0 2C	1.58	
0 3C	4.80			0 3C	2.18	
12 C1	4.66	6.02		12 C1	5.02	2.88
12 C2	6.40			12 C2	2.61	
12 C3	7.00			12 C3	1.00	
36 C 1	4.65	3.52		36 C 1	1.11	2.14
36 C 2	2.19			36 C 2	2.52	
36 C 3	3.72			36 C 3	2.79	
48 T 1	1.68	1.49		48 T 1	1.42	1.66
48 T 2	1.80			48 T 2	1.17	
48 T 3	1.00			48 T 3	2.38	
60 T 1	1.64	1.58		60 T 1	1.80	1.47
60 T 2	1.67			60 T 2	1.45	
60 T 3	1.43			60 T 3	1.16	
72 T 1	1.59	2.36		72 T 1	3.44	2.73
72 T 2	2.26			72 T 2	3.12	
72 T 3	3.22			72 T 3	1.62	
78 T 1	2.22	2.88		78 T 1	1.39	4.37
78 T 2	2.18			78 T 2	4.38	78 T1 outlier
78 T 3	4.25			78 T 3	4.36	
84 T 1	6.68	5.65		84 T 1	2.71	3.44
84 T 2	5.85			84 T 2	2.33	
84 T 3	4.43			84 T 3	5.29	

YP314a1 Treatment		
Identifier	Expression	Average
0 1C	7.90	8.33
0 2C	9.90	
0 3C	7.19	
12 C1	7.73	8.07
12 C2	7.11	
12 C3	9.38	
36 C 1	16.93	13.01
36 C 2	8.14	
36 C 3	13.97	
48 T 1	8.66	8.63
48 T 2	9.24	
48 T 3	8.01	
60 T 1	5.63	6.33
60 T 2	6.71	
60 T 3	6.64	
72 T 1	1.00	4.07
72 T 2	4.61	
72 T 3	6.59	
78 T 1	5.14	5.78
78 T 2	5.80	
78 T 3	6.39	
84 T 1	5.82	6.40
84 T 2	5.45	
84 T 3	7.94	

CYP18a1 Treatment		
Identifier	Expression	Average
0 1C	6.63	6.60
0 2C	4.55	outlier
0 3C	6.57	
12 C1	2.84	3.49
12 C2	3.75	
12 C3	3.87	
36 C 1	1.70	1.64
36 C 2	1.00	
36 C 3	2.20	
48 T 1	2.29	1.83
48 T 2	1.54	
48 T 3	1.67	
60 T 1	2.80	3.06
60 T 2	2.48	
60 T 3	3.91	
72 T 1	11.51	8.98
72 T 2	6.96	
72 T 3	8.47	
78 T 1	28.72	16.62
78 T 2	15.47	
78 T 3	5.69	
84 T 1	10.76	8.34
84 T 2	9.23	
84 T 3	5.04	

Fig 4. The influence of sodium nitroprusside on the duration of the molt cycle.

Control = animals not exposed to SNP
Treatment = animals exposed to SNP at 36 hours post molt

Control	
number	time between molts
1	72
2	72
3	74
4	70
5	74
6	74
7	72
8	74
9	84
10	72
11	84

Treatment	
number	time between molts
1	74
2	84
3	76
4	88
5	78
6	88
7	78
8	76
9	84
10	88
11	74

Fig 5. *E75, HR3, FTZ-F1, CYP18a1* mRNA levels with E75 dsRNA feeding.

Control= animals fed empty vector, E75= animals fed bacteria containing dsRNA targeting E75
Combined results from 4 experiments (March 2017, September 2017, October 2017, and July 2018).
Each data point is normalized to the mean mRNA expression of the controls for that experiment.

	E75		Mean	HR3		Mean	FTZ		Mean	CYP18a1		Mean
	Control	E75	Control	Control	E75	Control	Control	E75	Control	Control	E75	Control
Mar-17	169.17	23.17	89.12333	230.16	16.3	120.293333	1.15	3.75	2.52667	1.41	1	4.82
	77.19	53.18		43.63	173.33		5.43	3.04		7.82	1	
	21.01	57.85		87.09	87.74		1	8.03		5.23	1.02	
Sep-17	22.43	21.49	27.5625	2.7	2.92	1.5925	1	1.64	1.925	12.15	4.35	12.15
	21.11	1.18		1	1.96		1.58	1.21		12.95	2.45	
	37	1		1.42	1.71		1.15	1.78		13.59	1	
	29.71	24.62		1.25	2.66		3.97	4.22		9.91	4.34	
Oct-17	5.13	3.7	9.528	1	1.75	1.208	1	3.61	3.09	2	1	3.122
	14.16	7.19		1.22	1.89		7.11	2.91		4.5	1.9	
	9.24	4.5		1.4	1.74		2.73	6.29		3.12	1.63	
	11.83	5.17		1.02	2.37		2.38	5.07		2.94	2.47	
	7.28	5.4		1.4	2.55		2.23	10.78		3.05	2.11	
Jul-18	2.58	3.04	3.54	1.89	1.48	1.9075	2.02	2	1.2625	7.45	6.39	7.61
	4.16	4.19		2.23	1.47		1	2.29		8.63	4.1	
	3.22	2.67		1	2.06		1	3.05		4.98	2.88	
	4.2	1		2.51	2.86		1.03	2.97		9.38	1	

Normalization

E75		HR3		FTZ		Cyp18	
Control	E75	Control	E75	Control	E75	Control	E75
1.898156	0.100669	1.913323	0.135502	0.455145	1.484167	0.292531	0.207469
0.866103	0.231057	0.362697	1.440894	2.149074	1.203165	1.622407	0.207469
0.235741	0.251347	0.72398	0.729384	0.395778	3.178096	1.085062	0.211618
0.813787	0.779683	1.695447	1.833595	0.519481	0.851948	1	0.358025
0.765896	0.042812	0.627943	1.230769	0.820779	0.628571	1.065844	0.201646
1.342404	0.036281	0.89168	1.073783	0.597403	0.924675	1.118519	0.082305
1.077914	0.893243	0.784929	1.67033	2.062338	2.192208	0.815638	0.357202
0.538413	0.388329	0.827815	1.448675	0.323625	1.168285	0.640615	0.320307
1.486146	0.754618	1.009934	1.56457	2.300971	0.941748	1.441384	0.608584
0.969773	0.472292	1.15894	1.440397	0.883495	2.035599	0.999359	0.522101
1.241604	0.542611	0.844371	1.961921	0.770227	1.640777	0.941704	0.79116
0.764064	0.566751	1.15894	2.110927	0.721683	3.488673	0.976938	0.675849
0.728814	0.858757	0.990826	0.775885	1.6	1.584158	0.978975	0.839685
1.175141	1.183616	1.169069	0.770642	0.792079	1.813861	1.134034	0.538765
0.909605	0.754237	0.524246	1.079948	0.792079	2.415842	0.654402	0.378449
1.186441	0.282486	1.315858	1.499345	0.815842	2.352475	1.232589	0.131406

Fig 6. Impact of E75 suppression on (A) the duration of the molt cycle, (B) fecundity of maternal daphnids, and (C) incidence of developmental abnormalities among offspring.

Figure 6A

Molt Duration (hours) following treatment with empty vector or dsRNA targeting E75

EV	E75
80	79
76	84
76	78
80	80
80	80
76	80
78	81
76	84
80	84
76	81
80	82
82	84

*Note: EV (empty vector) = control; E75= E75 knockdown via feeding bacteria expressing dsRNA targeting E75

Figure 6B

Total neonates born in 2 broods. Mean + SEM in Fig 6B

Mom	EV	E75
1	16	22
2	20	17
3	23	13
4	20	21
5	20	23
6	19	24
7	23	21
8	31	19
9	18	26
10	20	26
11	20	14
12	22	19
13	19	3
14	30	23
15	31	12
16	32	29
17	28	20
18	30	21
19	21	13
20	21	

*Note: EV (empty vector) = control; E75= E75 knockdown via feeding bacteria expressing dsRNA targeting E75

Figure 6C

Abnormal development following treatment with empty vector or dsRNA targeting E75

*Note: EV (empty vector) = control; E75= E75 knockdown via feeding bacteria expressing dsRNA targeting E75

Ab = # Abnormal neonates in the brood

	EV				E75			
Mom	Brood 1	Ab	Brood 2	Ab	Brood 1	Ab	Brood 2	Ab
1	8		8		9		13	
2	12		8		8		9	
3	7		16	1	2		11	1
4	11		9		10		11	
5	5		15		8		15	
6	9		10		11		13	
7	11		12		11		10	
8	16		15		11		8	
9	9		9		11		15	
10	10		10		9		17	1
11	9		11	1	4		10	
12	14		8		6		13	
13	11		8		1		2	
14	10		20		13		10	1
15	12	1	19	1	4		8	
16	13		19		14		15	
17	9		19		11		9	
18	14		16		10	1	11	
19	10		11		2		11	
20	9		12					
Total/brood	209		255		155		211	
Total neonates in 2 broods	464				366			
Total number abnormal	4				4			
**Total percentage abnormal**	**0.862069**				**1.092896**			
